# Hyperglycemia During Acute Spinal Cord Injury Is a Detrimental Factor That Impairs Functional Improvement in Acute C3-C4 Cervical Cord Injury Patients Without Any Bony Damages

**DOI:** 10.1186/2197-425X-3-S1-A33

**Published:** 2015-10-01

**Authors:** K Kubota, K Kobayakaya, S Okada, K Shiba, Y Iwamoto

**Affiliations:** Kyushu University Hospital, Emergency & Critical Care Center, Fukuoka, Japan; Graduate School of Medical Sciences, Kyushu University, Departments of Orthopaedic Surgery, Fukuoka, Japan; Graduate School of Medical Sciences, Kyushu University, Departments of Advanced Medical Initiatives, Fukuoka, Japan; Japan Labour Health and Welfare Organization Spinal Injuries Center,, Department of Orthopaedic Surgery, Iizuka, Japan

## Introduction

Spinal cord injury (SCI) is a major public health problem and a devastating event for individuals. Because the central nervous system has a limited capacity for endogenous regeneration and repair, it is necessary to identify factors that exacerbate SCI to prevent any further deterioration of neurological function and improve the outcomes of injuries[1]. We previously demonstrated that transient hyperglycemia during acute SCI is a detrimental factor that impairs functional improvement in mice and human patients after acute SCI[2]. Although we performed not only Pearson x^2^ analysis but also a multiple linear regression analysis in the previous study, neurological function is difficult to be assessed for the patients with multiple injury levels. It is more ideal to estimate neurological recovery after SCI to match the injury level.

## Objective

To clarify the effects of hyperglycemia on the functional outcomes after SCI.

## Methods

To assess whether hyperglycemia purely affects neurologic outcomes of the patients with the same injury level, in this study, we selected acute C3-C4 cervical cord injury patients without any bony damages, which is most popular SCI in Japan. We also eliminated the patients with surgical treatments to exclude the surgical effect on neurological recovery. We retrospectively identified 48 SCI patients admitted within 24 hours after injury to the Spinal Injuries Center between June 2005 and May 2011. The blood samples of the patients were corrected immediately after being transported to our hospital (within 24 hours after injury), and the blood glucose concentration was measured in each sample. We examined the relationships between the admission blood glucose concentration values and other functional/clinical measurements, including American Spinal Injury Association (ASIA) Impairment Scale (AIS) grade, and the total spinal cord independence measure (SCIM) scores (for ADLs) at the final follow-up.

## Results

Pearson x^2^ analysis of data for 48 patients with SCI indicated that hyperglycemia on admission (glucose concentration ≧ 126 mg/dl) was a significant risk predictor of poor functional outcome.

## Conclusions

We showed that hyperglycemia during acute SCI may be a useful prognostic factor with a negative impact on motor function, highlighting the importance of achieving tight glycemic control after central nervous system injury.

## Grant Ackowledgement

This work was supported by JSPS KAKENHI Grant Number 26861198.

## References

[1] Kumamaru H (2012). Direct isolation and RNA-seq reveal environment-dependent properties of engrafted neural stem/progenitor cells. Nat Commun.

[2] Kobayakawa K (2014). Acute hyperglycemia impairs functional improvement after spinal cord injury in mice and humans. Sci Transl Med.

